# Pro-Apoptotic and Anti-Migration Properties of a Thiazoline-Containing Platinum(II) Complex in MDA-MB-231 Breast Cancer Cells: The Role of Melatonin as a Synergistic Agent

**DOI:** 10.3390/antiox11101971

**Published:** 2022-10-01

**Authors:** Samuel Estirado, Elena Fernández-Delgado, Emilio Viñuelas-Zahínos, Francisco Luna-Giles, Ana B. Rodríguez, José A. Pariente, Javier Espino

**Affiliations:** 1 Neuroimmunophysiology and Chrononutrition Research Group, Department of Physiology, Faculty of Science, University of Extremadura, 06006 Badajoz, Spain; 2 Coordination Chemistry Research Group, Department of Organic and Inorganic Chemistry, Faculty of Science, University of Extremadura, 06006 Badajoz, Spain

**Keywords:** melatonin, metallodrugs, platinum(II) complex, thiazoline, apoptosis, breast cancer

## Abstract

Triple-negative breast cancer (TNBC) is an aggressive cancer insensitive to hormonal and human epidermal growth factor receptor 2 (HER2)-targeted therapies and has a poor prognosis. Therefore, there is a need for the development of convenient anticancer strategies for the management of TNBC. In this paper, we evaluate the antitumoral potential of a platinum(II) complex coordinated with the ligand 2-(3,5-diphenylpyrazol-1-yl)-2-thiazoline (DPhPzTn), hereafter PtDPhPzTn, against the TNBC cell line MDA-MB-231, and compared its effect with both cisplatin and its less lipophilic counterpart PtPzTn, the latter containing the ligand 2-(pyrazol-1-yl)-2-thiazoline (PzTn). Then, the putative potentiating actions of melatonin, a naturally occurring antioxidant with renowned antitumor properties, on the tumor-killing ability of PtDPhPzTn were also checked in TNBC cells. Our results show that PtDPhPzTn presented enhanced cytotoxicity compared to both the classical drug cisplatin and PtPzTn. In addition, PtDPhPzTn was able to induce apoptosis, being more selective for MDA-MB-231 cells when compared to non-tumor breast epithelial MCF10A cells. Likewise, PtDPhPzTn produced moderate S phase arrest and greatly impaired the migration ability of MDA-MB-231 cells. Most importantly, the co-stimulation of TNBC cells with PtDPhPzTn and melatonin substantially enhanced apoptosis and markedly improved the anti-migratory action compared to PtDPhPzTn alone. Altogether, our findings provide evidence that PtDPhPzTn and melatonin could be potentially applied to breast cancer treatment as powerful synergistic agents.

## 1. Introduction

Triple-negative breast cancer (TNBC) is the least common type of breast cancers, accounting for 10–15% of all cases, but is often more aggressive and difficult to treat due to the lack of efficient molecular targets, such as estrogen receptor (ER), progesterone receptor (PR), and human epidermal growth factor receptor 2 (HER2) [1]. Chemotherapy is one of the most widely used therapeutic modalities in cancer treatment, which includes a wide variety of drugs. Chemotherapeutic agents induce apoptotic cell death not only in tumor cells but also in normal non-tumor cells, thus producing both therapeutic effects (i.e., killing tumor cells) and undesirable side effects (e.g., nephrotoxicity and cardiotoxicity).

Most metal-based antitumor therapies, such as *cis*-diamminedichloroplatinum(II) (cisplatin), have been successfully employed for more than 40 years. However, the use of cisplatin may be limited by the intrinsic or acquired resistance of various types of cancer and its toxic side effects. With the idea of overcoming limitations of cisplatin therapy, platinum metallodrugs with a similar structure to cisplatin have been synthesized in recent years. To design new platinum(II) anticancer drugs, several structural characteristics can be modified by changing the nature of the ligands bound to platinum(II), which influences its chemical and biological properties. For instance, platinum(II) complexes have been synthesized with bioactive bidentate ligands derived from heterocyclic N-donors, such as the heterocycles of pyrazole [2] and thiazoline [3], which have shown promising antitumor properties and may lay the foundation for designing new ligands that coordinate the platinum(II) ion. In this sense, our research group has recently synthesized complexes of platinum(II) with ligands of this nature that reportedly produced cytotoxic effects in human leukemic HL-60 [4], histiocytic lymphoma U-937 [5], ovarian adenocarcinoma SK-OV-3, and cervical epithelial HeLa cells [6,7], these effects being dependent on apoptosis induction, caspase cascade activation, increased intracellular reactive oxygen species (ROS) generation, and/or DNA oxidative damage [4,5,7].

Melatonin (*N*-acetyl-5-methoxytryptamine) is a neurohormone mainly produced by the pineal gland that plays a crucial role in regulating cancer growth, especially in hormone-dependent breast tumors [8]. Melatonin applies its anticancer abilities through multiple mechanisms, including promoting apoptosis and/or autophagy, modulation of angiogenesis [9], and selective induction of oxidative stress due to its pro-oxidant effects in cancer cells [10,11]. Of note, there are various aspects of melatonin research on cancer treatment that could have immediate clinical applications. For instance, many studies have shown that melatonin administered together with conventional drugs (e.g., cisplatin, 5-fluorouracil, and doxorubicin) enhances the sensitivity of cancers (e.g., colorectal, cervix, and pancreatic) to the induction of cell death [12,13,14,15,16,17]. Melatonin is also able to interfere with tumor metastases in a variety of cancers by employing multiple mechanisms, such as epithelial–mesenchymal transition, cytoskeleton reorganization, and extracellular matrix remodeling [18]. Furthermore, indoleamine causes treatment-resistant malignancies to become susceptible to both endocrine therapy and chemotherapy [19,20]. In addition, melatonin’s ability to reduce the side effects of cancer therapies also merits further attention as indoleamine has been shown to mitigate cell damage in numerous experimental paradigms, e.g., oral mucositis due to ionizing radiation [21] or the cardio-hepatic and renal toxicity of different drugs [22]. Therefore, mounting evidence suggests the convenience of using melatonin as adjuvant therapy for cancer treatment, which would far exceed improvements in the wellbeing of the patients.

Building upon previous data from our research group indicating that the enhanced lipophilicity of metallodrugs resulted in an improved ability to kill tumor cells [7], we evaluate in this paper the antitumoral potential of a recently synthesized platinum(II) complex containing the ligand 2-(3,5-diphenylpyrazol-1-yl)-2-thiazoline (DPhPzTn), hereafter PtDPhPzTn, against the TNBC cell line MDA-MB-231, and compare its effect with the classical chemotherapeutic agent cisplatin and its less lipophilic counterpart PtPzTn, the latter containing the ligand 2-(pyrazol-1-yl)-2-thiazoline (PzTn). In addition, we hypothesize that the anti-proliferative and pro-oxidant properties of melatonin could potentiate the tumor-killing ability of PtDPhPzTn in MDA-MB-231 cells.

## 2. Materials and Methods

### 2.1. General Procedures

The human epithelial breast adenocarcinoma MDA-MB-231 cell line, human histiocytic lymphoma U-937 cell line (No. 85011440), human promyelocytic leukemia HL-60 15–12 cell line (No. 88120805), and human epithelial cervix adenocarcinoma HeLa cell line (No. 93021013) were acquired from the European Collection of Authenticated Cell Cultures (ECACC) (Dorset, UK). The human mammary epithelial MCF10A cell line (No. CRL-10317) was obtained from the American Type Culture Collection (ATCC) (Barcelona, Spain). Roswell Park Memorial Institute (RPMI)-1640, Dulbecco’s modified Eagle’s medium (DMEM), DMEM F-12, penicillin/streptomycin, fetal bovine serum (FBS), and L-glutamine were purchased from ThermoFisher Scientific (Barcelona, Spain). *cis*-Diamminedichloroplatinum(II) (cisplatin), melatonin, hydrocortisone, insulin, epidermal growth factor (EGF), and cholera toxin were bought from Sigma Aldrich (Madrid, Spain). All other reagents were of analytical/commercial grade and were used without further purification. The ligands PzTn and DPhPzTn [23,24] and the precursor [PtCl_2_(DMSO)_2_] [25] were synthesized as described in the literature.

For the elemental analysis, a Leco CHNS-932 microanalyzer was used. IR spectra were recorded on a Perkin-Elmer 100 FTIR spectrophotometer from KBr pellets in the 4000–400 cm^−1^ range. ^1^H NMR spectra were obtained with a Bruker Avance III 500 instrument at 500 MHz in DMF-d7. ^1^H NMR signals were referenced to residual proton resonances in deuterated solvents.

### 2.2. General Preparation of Platinum(II) Complexes

**Scheme 1:**
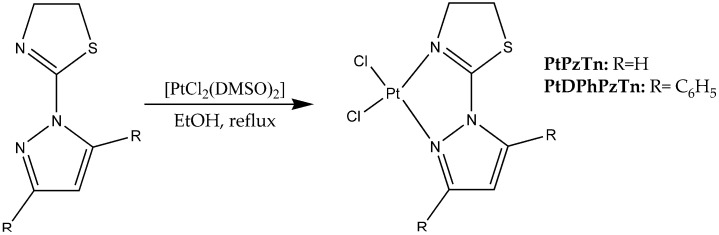
Synthesis of PtPzTn and PtDPhPzTn.

To perform the synthesis (Scheme 1), (300.0 mg, 0.71 mmol) the [PtCl_2_(DMSO)_2_] precursor was dissolved in 30 mL of ethanol in an Elenmeyer flask. On the other hand, the ligand was dissolved in 20 mL of ethanol. Once dissolved, the ligand corresponding was added to the precursor and it was refluxed and stirred for 24 h in the dark. The powder formed was vacuum filtered off and then recrystallized in DMF (for PtPzTn) or acetonitrile (for PtDPhPzTn). After that, crystals were separated by filtration, washed with distilled water and cold diethyl ether, and air-dried.

#### 2.2.1. Synthesis of [PtCl_2_(PzTn)] (PtPzTn)

This compound was obtained by the general procedure using PzTn (109 mg, 0.71 mmol). Yield 240.2 mg (80.6%). Anal. Calc. (%) for C_6_H_7_Cl_2_PtN_3_S: C, 17.19; H, 1.68; N, 10.02; S, 7.65%. Found: C, 17.41; H, 1.91; N, 9.80; S, 7.59%. IR (KBr): 3137, 3094, 2934, 1596, 1518, 1439, 1413, 1359, 1312, 1131, 1071, 1004, 781, 601 cm^−1^. ^1^H NMR (500 MHz, DMF-d7) δ 9.08 (d, 1H, J = 3.0 Hz), 8.29 (d, 1H, J = 2.5 Hz), 7.11 (t, 1H, J = 2.8 Hz), 4.47 (t, 2H, J = 8.5 Hz), 4.20 (t, 2H, J = 8.3 Hz), ppm.

#### 2.2.2. Synthesis of [PtCl_2_(DPhPzTn)] (PtDPhPzTn)

This compound was obtained by the general procedure using DPhPzTn (227 mg, 0.71 mmol). Yield 243.5 mg (59.9%). Anal. Calc. (%) for C_18_H_15_Cl_2_N_3_PtS: C, 37.84; H, 2.65; N, 7.35; S, 5.61%. Found: C, 37.78; H, 2.57; N, 7.45; S, 5.45%. IR (KBr): 3095, 3053, 1603, 1587, 1555, 1485, 1445, 1381, 1319, 1018, 998, 833, 768, 699 cm^−1^. ^1^H NMR (500 MHz, DMF-d7) δ 7.86 (m, 4H), 7.69 (m, 3H), 7.49 (m, 3H), 7.19 (d, 1H, J = 1.0 Hz), 4.42 (t, 2H, J = 8.5 Hz), 3.87 (t, 2H, J = 8.5 Hz) ppm.

### 2.3. Cell Culture and Treatments

The cell lines displayed in Table 1 were cultured into 25 cm^2^ culture flasks with the indicated media and supplements. Cells were kept under a humidified atmosphere containing 5% CO_2_ at 37 °C, and their viability was routinely tested by the Trypan-blue exclusion method. Cells were challenged with the platinum(II) complexes PtDPhPzTn and PtPzTn, the reference drug cisplatin, melatonin, or vehicle for 24 h at the indicated concentrations. Dimethylformamide (DMF) or dimethylsulfoxide (DMSO) were utilized as vehicle and its final concentration did not exceed 0.2% (*v*/*v*).

### 2.4. Cytotoxicity Assay

To examine the cytotoxic effects of the drugs on the different cell lines, the MTS method was performed by means of the CellTiter 96 AQueous One Solution Cell Proliferation assay (Promega, Madrid, Spain), as previously reported [6,7]. The experiments were run in triplicate and cells were challenged with the different drugs for 24 h.

Additionally, the effects (% cell death) of individual drugs and drug combinations were used to calculate combination index (CI) by using Compusyn Software (ComboSyn, Inc., Paramus, NJ, USA), following the Chou-Talalay method [26]. CI values indicate how the selected drug combinations influence their therapeutic efficacy (CI > 1–antagonistic; CI = 1–additive; CI < 1–synergistic) at different combination dosing intervals [27].

### 2.5. Determination of Apoptotic Cells

The population of apoptotic cells was studied by analyzing phosphatidylserine externalization via annexin V-FITC/propidium iodide (PI) assay (ThermoFisher Scientific, Barcelona, Spain), as previously described [14]. The percentages of live, early apoptotic, late apoptotic, and secondary necrotic cells were computed.

### 2.6. Caspase-3 and -9 Analysis

Caspase-3 and -9 activation was measured with CaspGLOW fluorescein active caspase-3 staining kit and Caspase-9 staining kit (Red), respectively, as published elsewhere [4]. The analysis of caspase activation was restricted to live cells, which were detected by using the vital dye Hoechst 33258 (10 μg/mL). Data are presented as the percentage of caspase-positive cells.

### 2.7. Measurement of Reactive Oxygen Species (ROS)

Reactive oxygen species (ROS) detection was performed by incubating cells (30 min, 37 °C) with 0.4 μM 2′,7′-dichlorodihydrofluorescein diacetate (DCFH-DA), as previously reported [4]. Again, the analysis of ROS production was restricted to live cells, which were detected by using the vital dye Hoechst 33258 (10 μg/mL). Data are portrayed as the percentage of DCF-stained cells.

### 2.8. Cell Cycle Analysis

To investigate changes in cell cycle progression upon treatment, a cell cycle distribution analysis was carried out in ethanol-fixed, PI-stained cells, as published elsewhere [4]. The populations of cells in G0/G1 transition, S phase, and G2/M transition, as well as sub-G1 (hypodiploid) cells are reported.

### 2.9. Wound-Healing Assay

Cells were seeded into 24-well plates and incubated at 37 °C until they reached 75–80% confluency. To evaluate cell migration, cells were equally wounded with a sterile 100 μL pipette tip and were subsequently challenged with the different treatments. The experiments were run in duplicate. The relative cell migration rates over 12 and 24 h were quantified based on the changes in wound area at time 0 h and after 12 or 24 h using an epifluorescence microscope (Nikon Eclipse TS100). The images were analyzed with the plugin “Wound healing size tool” available in Fiji/ImageJ software version 1.49 (https://github.com/AlejandraArnedo/Wound-healing-size-tool/wiki, accessed on 1 July 2022) [28].

### 2.10. Transwell Migration Assay

To assess cell migration, transwell migration assay was carried out in transwell chambers with non-coated membrane (24-well insert, pore size: 8 mm, Millipore, Madrid, Spain). Cells (1 × 10^5^) were plated in serum free medium in the upper chamber. The medium containing 10% FBS was added in the lower chamber to act as chemoattractant. After 24 h, the cells that migrated through the pores to the lower surface of the inserts were fixed in 70% ethanol solution for 10 min and afterwards stained with crystal violet for additional 10 min. Inserts were then imaged and, subsequently, the membranes of the inserts were cut, placed in a 24-well plate, and incubated with 5% sodium dodecyl sulfate (SDS) to extract crystal violet (5 min on orbital shaker). Finally, the absorbance was read at 560 nm for quantification.

### 2.11. Statistical Analysis

Data represent the mean ± standard deviation (S.D.). To compare between treatments, statistical significance was calculated by a one-way analysis of variance (ANOVA) followed by Dunnett’s test, unless otherwise specified. The half maximal inhibitory concentration (IC_50_) values of the dose–response curves were calculated by using nonlinear regression. *p* < 0.05 was considered to indicate a statistically significant difference. The statistics software used was GraphPad Prism 7.04 for Windows (GraphPad Software, San Diego, CA, USA).

## 3. Results and Discussion

**Figure 1:**
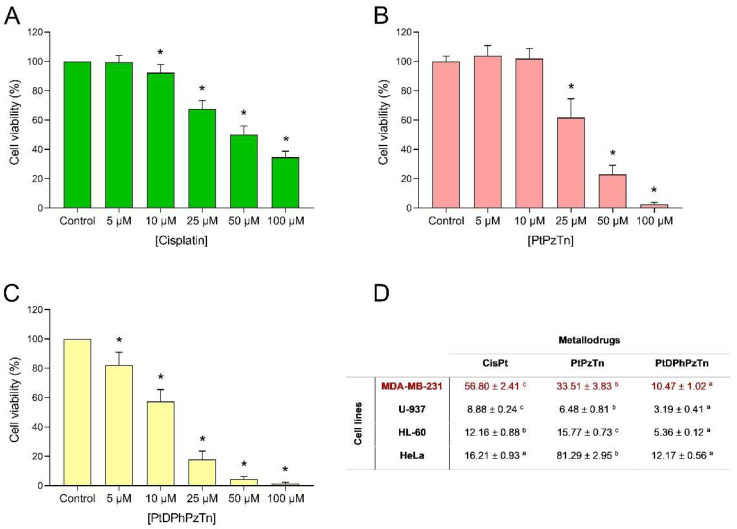
Dose–response curves of metallodrugs on cell viability. MDA-MB-231 cells were treated for 24 h with increasing concentrations (0–100 μM, as indicated) of cisplatin (**A**), the platinum(II) complexes PtPzTn (**B**) and PtDPhPzTn (**C**), or the vehicle (DMF, control). Data represent means ± S.D. of 6 independent experiments and are expressed as percentage of control values. * *p* < 0.05 compared to their corresponding control values (Dunnett’s test). (**D**) IC_50_ values (mean ± S.D., μM) of cisplatin (CisPt), PtPzTn, and PtDPhPzTn against MDA-MB-231, U-937, HL-60, and HeLa cell lines. Within each row, values followed by a diverse lowercase letter (a–c) are significantly different (*p* < 0.05; Tukey’s test).

The biological activity of a thiazoline-containing platinum(II) complex PtDPhPzTn was first assessed. As depicted in Figure 1C, PtDPhPzTn exhibited a dose-dependent cytotoxic effect against the TNBC cell line MDA-MB-231 with an IC_50_ value of 10.47 ± 1.02 μM (Figure 1D), which was more than 5-fold lower than that of the reference drug cisplatin (56.80 ± 2.41 μM, Figure 1A,D) and more than 3-fold lower than that of its less lipophilic counterpart PtPzTn (33.51 ± 3.83 μM, Figure 1B,D). The same trend was observed when the metallodrugs were tested in other cell lines, namely U-937, HL-60, and HeLa (Figure 1D), thereby demonstrating that PtDPhPzTn outperforms the reference drug cisplatin (and PtPzTn) in terms of cytotoxicity, though this fact was especially striking in the case of MDA-MB-231 cells, which were moderately resistant to cisplatin (IC_50_ > 50 µM). These findings agree with our previous research [7], thus indicating that the incorporation of aromatic groups in the ligand can lead to the improved cytotoxicity of metallodrugs.

**Figure 2:**
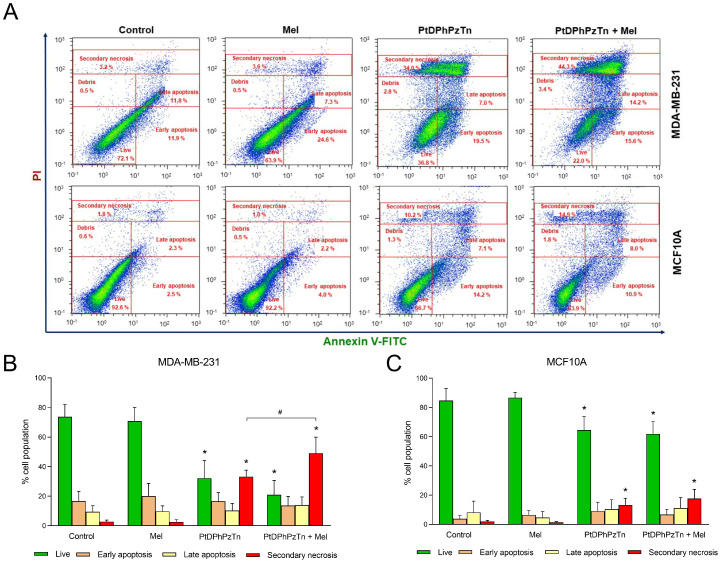
Combinatory treatment of PtDPhPzTn and melatonin differentially affects apoptosis of MDA-MB-231 and MCF10A cell lines. Cells were treated with 10.4 µM PtDPhPzTn in the presence or absence of 1 mM melatonin (Mel), or the vehicle (DMF/DMSO, control) for 24 h. (**A**) Representative cytograms depicting the redistribution of phosphatidylserine (annexin V-FITC staining) in the presence of propidium iodide (PI) after challenging cells with the indicated drugs. Histogram bars show percentages of live, early apoptotic, late apoptotic, and secondary necrotic MDA-MB-231 (**B**) and MCF10A (**C**) cells. Values represent means ± S.D. of 5 independent experiments. * *p* < 0.05 compared to control values (Dunnett’s test). ^#^ *p* < 0.05 compared to PtDPhPzTn-treated cells (Tukey’s test).

Given that the platinum(II) complex PtDPhPzTn was the most effective drug in terms of cytotoxicity and the well-known anti-proliferative and pro-oxidant properties of the indoleamine melatonin in tumor cells, the apoptosis-promoting effect of the combinatory treatment with PtDPhPzTn and melatonin was determined in TNBC cells by analyzing phosphatidylserine externalization in the presence of PI. For this purpose, MDA-MB-231 cells were exposed at the fixed IC_50_ dose level of PtDPhPzTn (i.e., 10.4 µM) in the absence or presence of 1 mM melatonin. As shown in Figure 2, the platinum(II) complex caused a remarkable increase (33.25 ± 4.34% vs. 2.78 ± 1.04%; *p* < 0.05) in the population of secondary necrotic cells at the expense of a significant drop (32.13 ± 11.98% vs. 73.63 ± 8.38%; *p* < 0.05) in the proportion of live cells with respect to the control cells (Figure 2A,B), which is in line with our preliminary findings, indicating that the cytotoxic effect of PtDPhPzTn was associated with the induction of ROS-mediated apoptosis [7]. Previous studies have reported that pyrazole-platinum(II) complexes, especially those containing methyl substituents at the pyrazole ring, exhibited strong pro-apoptotic activity in MDA-MB-231 and MCF-7 breast cancer cell lines [29]. Likewise, dinuclear berenil-platinum(II) complexes were also described to trigger cellular oxidative modifications and, subsequently, apoptosis in MDA-MB-231 and MCF-7 breast cancer cell lines [30,31]. Melatonin per se did not produce any significant pro-apoptotic effect on MDA-MB-231 cells (Figure 2A,B). Interestingly, the combinatory treatment with PtDPhPzTn and melatonin further promoted apoptosis in TNBC cells, as demonstrated by a notable rise (49.11 ± 10.86% vs. 33.25 ± 4.34%; *p* < 0.05) in the percentage of secondary necrotic cells compared to the PtDPhPzTn-treated cells (Figure 2A,B). In this sense, melatonin has been previously shown to synergistically enhance antitumor function in tunicamycin-challenged MDA-MB-231 cells [32], which is in line with the results reported in this paper. The potentiating actions of melatonin on apoptosis induction by classical drugs, such as doxorubicin, lapatinib, and arsenic trioxide, has been also documented in breast cancer cell lines [33,34,35,36] and may be related to its pro-oxidant capacity in tumor cells [36]. To corroborate the combinatorial effect of PtDPhPzTn and melatonin in MDA-MB-231 cells, 5, 10, and 25 µM of the platinum(II) complex and 0.5, 1, and 2 mM of the indoleamine were combined and cell death was determined after 24 h to calculate the CI values (Figure S1). Our results show a synergistic effect of the combination of PtDPhPzTn and melatonin, especially when combining 1 mM melatonin and the three different concentrations of the platinum(II) complex (Table S1).

On the other hand, the complex PtDPhPzTn turned out to be less harmful to MCF-10A human breast epithelial cells than to tumor cells, as the very same dose of PtDPhPzTn (10.4 µM) only provoked a moderate reduction in the proportion of live cells (64.58 ± 9.31% vs. 84.76 ± 8.14%; *p* < 0.05; Figure 2A,C) with respect to control cells. Other platinum(II) complexes, including cyclometalated complexes and metal complexes with berenil and nitroimidazole, have been informed to show lower toxicity towards normal MCF-10A cells as well [37,38]. In addition, melatonin per se did not affect apoptosis of MCF10A cells (Figure 2A,C), which agrees with previous findings documenting no effect on nontumoral breast epithelial MCF10A cells [39,40]. Concerning the combinatory treatment, and contrary to what happened on MDA-MB-231 breast cancer cells, it did not display any potentiating effect on the PtDPhPzTn-induced apoptosis of MCF10A cells (Figure 2A,C), thus indicating that this combined treatment exhibits lower apoptosis-promoting actions on normal mammary epithelial cells than on breast cancer cells. 

**Figure 3:**
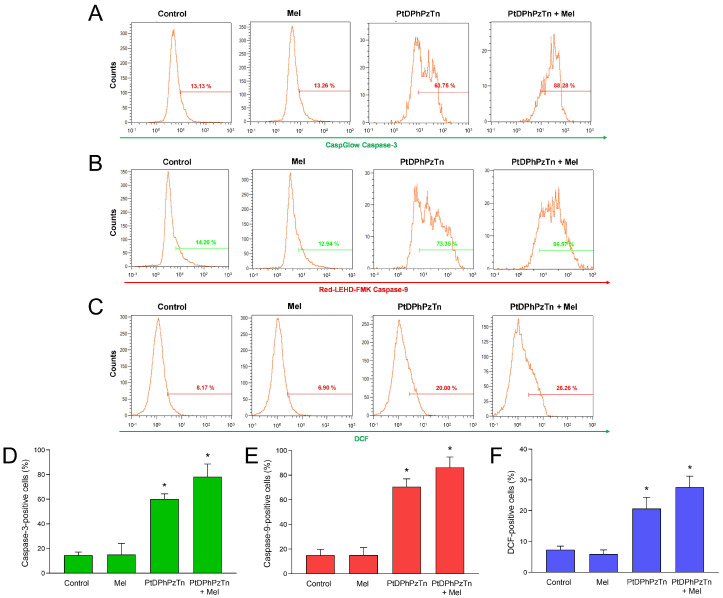
Caspase activation and reactive oxygen species (ROS) production triggered by combinatory treatment of PtDPhPzTn and melatonin in MDA-MB-231 cells. Cells were treated with 10.4 µM PtDPhPzTn in the presence or absence of 1 mM melatonin (Mel), or the vehicle (DMF/DMSO, control) for 24 h (except for ROS production, wherein cells were challenged for 4 h). Representative cytograms show caspase-3 (**A**) and -9 (**B**) activation as well as ROS production (**C**) after challenging cells with the indicated drugs. Histogram bars represent percentages of caspase-3-positive (**D**), caspase-9-positive (**E**), and DCF-stained (**F**) MDA-MB-231 cells, which were analyzed by flow cytometry (for details, see Section 2.6 and Section 2.7). Values are presented as means ± SD of four independent experiments. * *p* < 0.05 compared to control values (Dunnett’s test).

To confirm that the main mode of cell death elicited by the platinum(II) complex was apoptosis, the activation of caspases, which are enzymes critically involved in the initiation and execution of such process, was investigated. When MDA-MB-231 cells were treated with 10.4 µM PtDPhPzTn for 24 h, a remarkable rise in the proportion of caspase-3-positive (60.17 ± 4.27% vs. 14.57 ± 9.08%; *p* < 0.05; Figure 3A,D) and caspase-9-positive (70.63 ± 6.37% vs. 15.07 ± 4.67%; *p* < 0.05; Figure 3B,E) cells was noted, thus indicating that the platinum(II) complex triggered intrinsic apoptosis in the TNBC cell line. In this sense, earlier studies have informed that both pyrazole-platinum(II) complexes and dinuclear berenil-platinum(II) complexes also caused caspase-3 and caspase-9-dependent apoptosis in breast cancer cells [29,30,31]. Of note, the combinatorial treatment with PtDPhPzTn and melatonin moderately potentiated the activation of caspases, though this effect was not statistically significant compared to PtDPhPzTn alone (78.28 ± 10.30% vs. 60.17 ± 4.27% and 86.30 ± 8.43% vs. 70.63 ± 6.37% for caspase-3 and -9, respectively; Figure 3A,B,D,E). Again, melatonin per se produced negligible effect on caspase activation of MDA-MB-231 cells (Figure 3A,B,D,E). On the other hand, the involvement of ROS in the apoptotic process evoked by the platinum(II) complex was also explored. Our findings show that PtDPhPzTn notably increased intracellular ROS production after 4 h (20.70 ± 3.66 vs. 7.35 ± 1.15% DCF-positive cells; *p* < 0.05; Figure 3C,F), which ultimately led to apoptotic cell death. These results agree with previous research describing that other platinum(II)-based also exerted their cytotoxic effect in breast cancer cells through oxidative stress-mediated apoptosis [30]. Additionally, the combined treatment with PtDPhPzTn and melatonin further augmented intracellular ROS production, this effect not being statistically significant compared to PtDPhPzTn-treated cells (26.29 ± 5.39% vs. 20.70 ± 3.66 DCF-positive cells; *p* < 0.05; Figure 3C,F). As for melatonin alone, it did not induce any change in the ROS production of MDA-MB-231 cells (Figure 3C,F).

**Figure 4:**
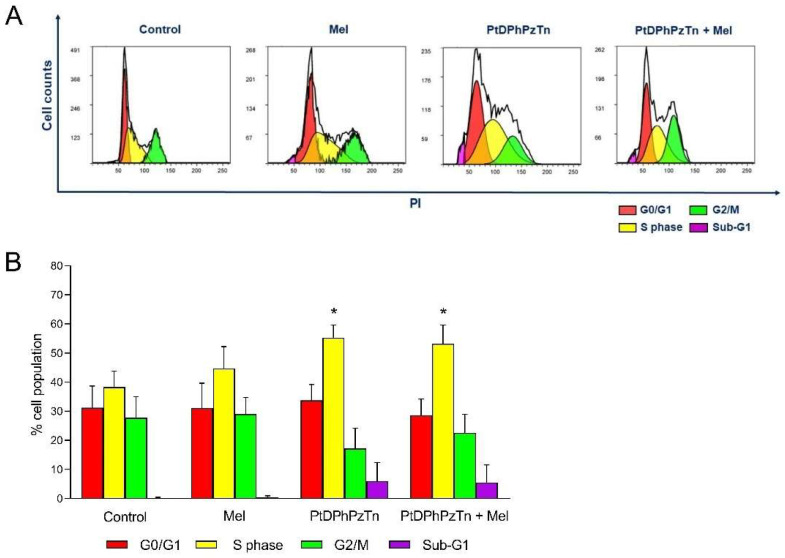
Combinatory treatment of PtDPhPzTn and melatonin disturbed cell cycle profile of MDA-MB-231 cell line. Cells were treated with 10.4 µM PtDPhPzTn in the presence or absence of 1 mM melatonin (Mel) or the vehicle (DMF/DMSO, control) for 24 h. (**A**) Representative cytograms displaying cell cycle distribution after challenging cells with the indicated drugs. (**B**) Histogram bars show percentages of cells in G0/G1 transition, S phase, G2/M transition and sub-G1 population (hypodiploid cells). Values represent means ± S.D. of 5 independent experiments. * *p* < 0.05 compared to control values (Dunnett’s test).

As the platinum(II) complex displayed pro-apoptotic actions against MDA-MB-231 cells, its impact on cell cycle progression was anticipated and, therefore, cell cycle distribution analysis was carried out. Thus, when TNBC cells were challenged with 10.4 µM PtDPhPzTn, a moderate S phase arrest (55.32 ± 4.28% vs. 38.30 ± 5.44% in control cells; *p* < 0.05; Figure 4) was observed at the expense of the percentage of cells in G2/M transition. Additionally, the platinum(II) complex caused a rise in the subpopulation of cells with hypodiploid DNA content (sub-G1 cells; Figure 4), which is indicative of DNA fragmentation. In this line, earlier studies have proven that cyclometalated platinum(II) complexes also affect S phase of the cell cycle in breast cancer cells [37]. Nonetheless, other investigations have demonstrated that the inhibition of cell survival induced by pyrazole-platinum(II) complexes occurs by arresting the G1 phase of the cell cycle [29]. The same trend was observed for the combinatory treatment, though no potentiating effect was noticed. In fact, when TNBC cells were treated with 10.4 µM PtDPhPzTn in the presence of 1 mM melatonin, a significant accumulation of MDA-MB-231 cells at S phase (53.18 ± 6.39% vs. 38.30 ± 5.44% in control cells; *p* < 0.05; Figure 4) was ascertained. Likewise, the co-incubation with the platinum(II) complex and the indoleamine evoked an increment in the percentage of cells with hypodiploid DNA content (Figure 4). Nevertheless, melatonin per se did not induce any remarkable change in the cell cycle distribution of MDA-MB-231 cells (Figure 4), which is in line with previous research indicating no effect of indoleamine on the cell cycle of breast cancer cells [41].

**Figure 5:**
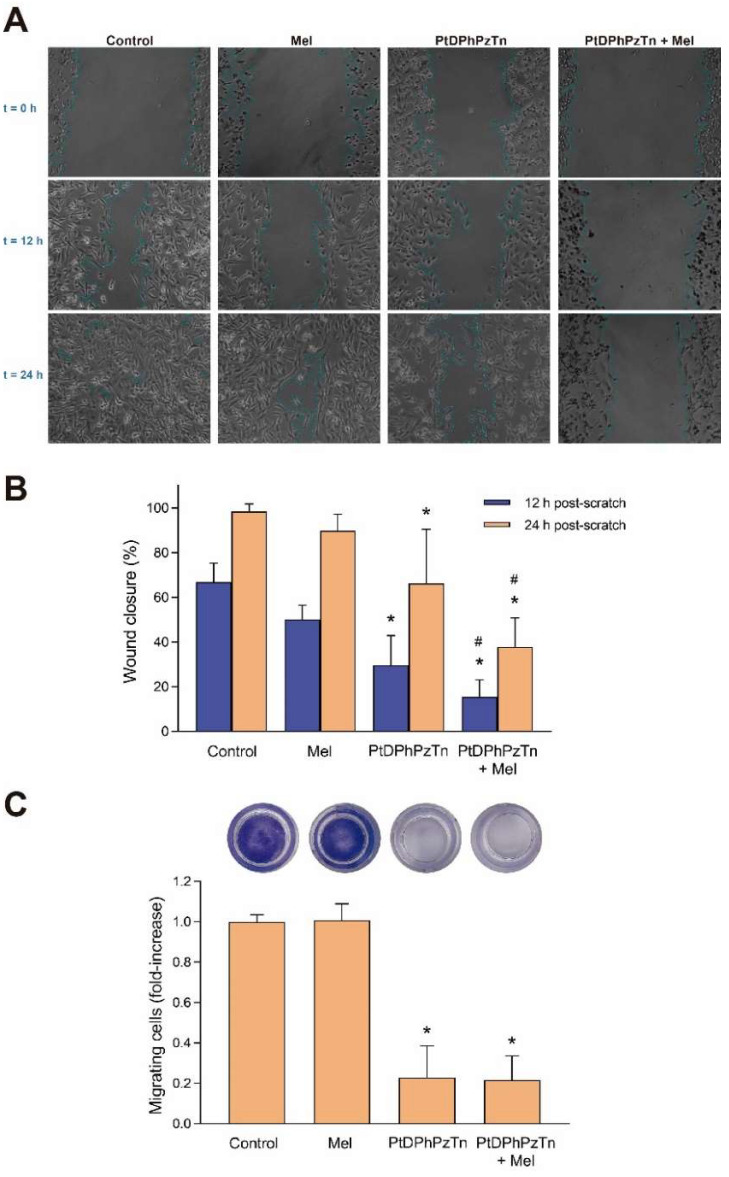
Combinatory treatment of PtDPhPzTn and melatonin altered migration properties of MDA-MB-231 cell line. Cells were treated with 10.4 µM PtDPhPzTn in the presence or absence of 1 mM melatonin (Mel) or the vehicle (DMF/DMSO, control) for 24 h. (**A**) Representative images depicting wound-healing assay in cells challenged with the indicated drugs. (**B**) Histogram bars show the percentage of wound closure after 12 and 24 h, where 100% represents a fully closed wound. Values represent means ± S.D. of 12 independent experiments. (**C**) Proportion of migrating cells after 24 h of exposure to the indicated drugs determined by transwell migration assay (for details, see Section 2.10). Representative images of transwell inserts for each condition are depicted in the upper part. Data represent means ± S.D. of 5 independent experiments and are expressed as fold-increase of control values. * *p* < 0.05 compared to its corresponding control value (Dunnett’s test). ^#^ *p* < 0.05 compared to its corresponding PtDPhPzTn value (Tukey’s test).

Finally, the ability of the platinum(II) complex PtDPhPzTn to modulate cell migration capacity of TNBC cells was analyzed by wound-healing and transwell migration assays. As shown in Figure 5A,B, the PtDPhPzTn complex greatly impaired the ability of MDA-MB-231 cells to migrate after both 12 h (29.76 ± 13.11% vs. 66.79 ± 8.40% in control cells; *p* < 0.05) and 24 h post-scratch (66.19 ± 24.20% vs. 98.44 ± 3.26% in control cells; *p* < 0.05), thus suggesting that this compound may be useful in blocking the metastatic migration of breast cancer cells. The same trend was observed in the transwell migration assay, wherein the platinum(II) complex almost completely blunted the migration capacity of MDA-MB-231 cells (*p* < 0.05; Figure 5C). In this sense, other authors have also synthesized metal complexes with potential to suppress the cell migration ability of TNBC cells, including *cis*-diphenyl pyridineamine platinum(II) complexes and platinum(II) complexes with tridentate ligands [42,43]. As for the combined treatment, the exposure of MDA-MB-231 cells at 10.4 µM PtDPhPzTn in the presence of 1 mM melatonin markedly enhanced the antimigration effect of the platinum(II) complex after both 12 h (15.57 ± 7.53% vs. 29.76 ± 13.11% in PtDPhPzTn-treated cells; *p* < 0.05; Figure 5A,B) and 24 h post-scratch (37.85 ± 12.93% vs. 66.19 ± 24.20% in PtDPhPzTn-treated cells; *p* < 0.05; Figure 5A,B). Regarding the transwell migration assay, the combinatory treatment diminished (*p* < 0.05) the proportion of migrating cells to the same extent as the platinum(II) complex alone (Figure 5C). Other studies has also proven that indoleamine synergistically improves the antimigration properties of apatinib, a tyrosine kinase inhibitor thought to inhibit angiogenesis, in breast cancer stem cells derived from MDA-MB-231 cells [44], whereas novel melatonin–tamoxifen drug conjugates were shown to effectively inhibit migration in different breast cancer cell lines, including tamoxifen-resistant cells [45]. Nonetheless, even though melatonin has been described to inhibit cell invasion potential in MDA-MB-231 and MCF-7 breast cancer cell lines [46], indoleamine per se only evoked a moderate, non-significant impairment in the ability of TNBC cells to migrate (Figure 5A,B) and showed no effect in the transwell migration assay.

## 4. Conclusions

Platinum(II)-based complexes are undoubtedly an interesting alternative to canonical chemotherapy agents as they can help avoiding drug resistance and off target toxicity by promoting the apoptosis of tumor cells via multiple mechanisms, including oxidative stress, mitochondrial injury, and DNA damage. In this paper, we described that the thiazoline-containing platinum(II) complex PtDPhPzTn, which reportedly induces oxidative stress and apoptosis in tumor cells [7], outperformed, by far, the reference drug cisplatin and its less lipophilic counterpart PtPzTn in terms of cytotoxicity against MDA-MB-231 cells. More importantly, the combinatory treatment with PtDPhPzTn and the renowned antioxidant melatonin potentiated the pro-apoptotic, cell cycle arrest and anti-migration properties of the platinum(II) complex alone against MDA-MB-231 breast cancer cells. Therefore, our findings provide evidence that a combined therapy with PtDPhPzTn and melatonin could be potentially applied to breast cancer management as powerful synergistic agents.

## Figures and Tables

**Table 1:** List of cell lines, cell culture media, and supplements.

Cell Lines	Disease/Tissue	Growth Medium	Supplements
MDA-MB-231	Human breast adenocarcinoma	DMEM	10% heat inactivated FBS 2 mM L-glutamine 100 U/mL penicillin/streptomycin
HeLa	Human cervix adenocarcinoma		
U-937	Human histiocytic lymphoma	RPMI-1640	
HL-60	Human promyelocytic leukemia		
MCF10A	Human mammary epithelial cells	DMEM F-12	5% horse serum 20 ng/mL EGF 10 µg/mL insulin 100 ng/mL cholera toxin 0.5 µg/mL hydrocortisone 100 U/mL penicillin/streptomycin

DMEM: Dulbecco’s modified Eagle’s medium; EGF: epidermal growth factor; FBS: fetal bovine serum; RPMI: Roswell Park Memorial Institute.

## Data Availability

The data are contained within this article and the Supplementary File.

## Supplementary Materials

The following are available online at https://www.mdpi.com/article/10.3390/antiox11101971/s1, Figure S1. Synergistic effect of the combination of PtDPhPzTn and melatonin in MDA-MB-231 cells, Table S1. Calculation of the combination index (CI) data for non-constant combination of PtDPhPzTn and melatonin in cell death induction.
