# Supplementary material for: Pro-Apoptotic and Anti-Migration Properties of a Thiazoline-Containing Platinum(II) Complex in MDA-MB-231 Breast Cancer Cells: The Role of Melatonin as a Synergistic Agent
Source: Antioxidants (Basel). 2022 Oct 1;11(10):1971. doi: 10.3390/antiox11101971 (PMC9598564; doi:10.3390/antiox11101971)
Supplement: Supplementary file 1 [file antioxidants-11-01971-s001.zip › antioxidants-1863482-supplementary.pdf]

Supplementary Material

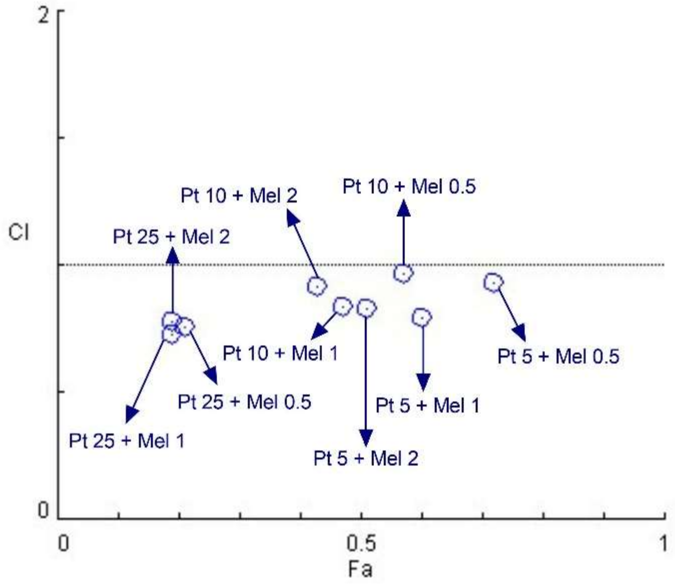

Figure S1. Synergistic effect of the combination of PtDPhPzTn and melatonin in MDA-MB-231 cells. It is shown an estimation plot of the combination treatment by Compusyn software with combination index (CI) values (y-axis) and fraction affected (Fa, x-axis). For the calculation, PtDPhPzTn (Pt; 5, 10 and 25 µM) and melatonin (Mel; 0.5, 1 and 2 mM) were used to induced cell death at the concentrations indicated for 24 h.

Table S1. Calculation of the combination index (CI) data for non-constant combination of PtDPhPzTn and melatonin in cell death induction.

PtDPhPzTn (µM)	Melatonin (mM)	Effect (Fa)	CI
5	0.5	0.72	0.93
10	0.5	0.57	0.96
25	0.5	0.21	0.75
5	1	0.60	0.79
10	1	0.47	0.83
25	1	0.19	0.72
5	2	0.51	0.83
10	2	0.45	0.91
25	2	0.19	0.77
